# Design and implementation of a massive open online course on enhancing the recruitment of minorities in clinical trials – Faster Together

**DOI:** 10.1186/s12874-021-01240-x

**Published:** 2021-03-05

**Authors:** Sheila V. Kusnoor, Victoria Villalta-Gil, Margo Michaels, Yvonne Joosten, Tiffany L. Israel, Marcia I. Epelbaum, Patricia Lee, Elizabeth T. Frakes, Jennifer Cunningham-Erves, Stephanie A. Mayers, Sarah C. Stallings, Nunzia B. Giuse, Paul A. Harris, Consuelo H. Wilkins

**Affiliations:** 1 Center for Knowledge Management, Strategy and Innovation, Vanderbilt University Medical Center, 3401 West End, Suite 304, Nashville, TN 37203 USA; 2 Meharry-Vanderbilt Alliance, Vanderbilt University Medical Center, Nashville, TN USA; 3 Health Action and Access Consulting, Boston, MA USA; 4 Boston University School of Public Health, Boston, MA USA; 5 Department of Medical Education and Administration, Vanderbilt University School of Medicine, Nashville, TN USA; 6 Vanderbilt Institute for Medicine and Public Health, Vanderbilt University Medical Center, Nashville, TN USA; 7 Department of Internal Medicine, Meharry Medical College, Nashville, TN USA; 8 Vanderbilt Institute for Clinical and Translational Research, Vanderbilt University Medical Center, Nashville, TN USA; 9 Department of Biomedical Informatics, Vanderbilt University Medical Center, Nashville, TN USA; 10 Department of Medicine, Vanderbilt University Medical Center, Nashville, TN USA; 11 Vice President for the Office of Health Equity, Vanderbilt University Medical Center, Nashville, TN USA

**Keywords:** Clinical trials, Education, Race, Ethnicity

## Abstract

**Background:**

Racial and ethnic minorities are often underrepresented in clinical trials, threatening the generalizability of trial results. Several factors may contribute to underrepresentation of minorities in clinical trials, including lack of training for researchers and staff on the importance of diversity in clinical trials and effective strategies for recruiting and retaining minority populations.

**Methods:**

Applying community engaged research principles, we developed a massive open online course (MOOC) to help research team members develop knowledge and skills to enhance the recruitment of minorities in clinical trials. A transdisciplinary working group, consisting of clinical researchers, community engagement specialists, minority clinical trial recruitment and retention educators and specialists, and knowledge management information scientists, was formed to develop an evidence-based curriculum. Feedback from the Recruitment Innovation Center Community Advisory Board was incorporated to help finalize the curriculum. The course was implemented in Coursera, an online learning platform offering MOOCs. A bootstrap paired sample t-test was used to compare pre- and post-assessments of knowledge, attitudes, and intentions as it relates to minority recruitment.

**Results:**

The final course, entitled Faster Together, was divided into eight 1-h modules. Each module included video presentations, reading assignments, and quizzes. After 10 months, 382 individuals enrolled in the course, 105 participants completed the pre-test, and 14 participants completed the post-test. Participants’ knowledge scores were higher with an increase in the mean number of correct answers from 15.4 (95% CI:12.1–18.7) on the pre-test to 18.7 (95% CI:17.42–20.2) on the post-test. All post-test respondents (*n* = 14) indicated that the course improved their professional knowledge, and 71.4% of respondents indicated that they were very likely to make changes to their recruitment practices.

**Conclusions:**

Faster Together, a massive open online course, is an acceptable, accessible approach to educating research teams on minority recruitment in clinical trials. Preliminary evidence indicates the course increased knowledge on how to recruit minorities into clinical trials and could promote change in their recruitment practices.

**Supplementary Information:**

The online version contains supplementary material available at 10.1186/s12874-021-01240-x.

## Background

Establishing diversity in clinical trials remains a research priority due to the challenges in recruiting and retaining racial and ethnic minorities [1–5]. Failure to achieve adequate diversity in research participation makes it difficult to understand population differences in the efficacy of treatments, address health disparities, and provide personalized, evidence-based medicine. Federal initiatives have been developed to address underrepresentation of minorities in clinical trials. For example, the 1993 National Institutes of Health (NIH) Revitalization Act [6] required the inclusion of women and minorities in clinical research studies receiving NIH funding. More recently, the U.S. Food and Drug Administration issued a draft guidance document to help sponsors increase accrual of underrepresented groups in clinical trials [7]. Yet, studies continue to demonstrate disparities in the recruitment of racial and ethnic minorities [3–5, 8, 9].

Underrepresentation of racial and ethnic minorities in clinical trials has been attributed to several different factors. Examples include distrust of researchers and clinicians, lack of culturally-appropriate educational materials about clinical trials, language barriers, immigration status concerns, and lack of knowledge about clinical trials [10–14]. Many of the strategies that have been proposed to increase minority participation in research aim at reducing the barriers to participation [15–17]. Recent findings, however, suggest that researchers and their teams may inadvertently contribute to underrepresentation through lack of knowledge about the importance of diversity in research participation and of skills to engage underrepresented groups. For example, Niranjan et al. [18] found that stakeholders, including principal investigators, research staff, referring clinicians, and cancer center leaders, from five U.S. cancer centers affiliated with the consortium for Enhancing Minority Participation in Clinical Trials (EMPaCT) were not receiving training on recruitment and retention of minority groups [18]. This highlights the need for comprehensive training to help researchers appreciate participant diversity and engage underrepresented minorities and marginalized communities in clinical trials.

The Recruitment Innovation Center (RIC) was funded in 2016 by the NIH to develop and foster evidence-based recruitment and retention practices for the Trials Innovation Network (TIN), a collaborative initiative aiming to optimize the infrastructure of clinical trials at a national level [19]. Addressing barriers to recruitment and retention in minority and underrepresented groups advances the overall RIC goals of improved clinical trials quality, increased and diversified trial enrollment, and improved national health outcomes. We developed a massive open online course (MOOC) aimed at providing training for research team members to develop skills and knowledge about the recruitment of minorities in clinical trials. Here, we describe our course development process and report the enrollment and course evaluation data in the 10-month period after the course was released.

## Methods

### Course development

A transdisciplinary working group was established to develop the course. The team included clinical researchers, community engagement specialists, minority clinical trial recruitment and retention educators and specialists, and knowledge management information scientists. To guide the curriculum development process, a review of the literature was conducted to identify: 1) knowledge and skills needed to effectively recruit and retain minorities in clinical trials; and 2) existing training programs and resources to help enhance recruitment and retention of minorities in clinical trials (Additional file 1). The search was not limited by study type. The results were summarized using a narrative format. See additional file 1 for a description of the methods and results from the literature review.

The working group applied their skills and expertise to the development of the course. The team was experienced with developing and testing innovative recruitment strategies that consider methods for gathering authentic community feedback [20], evidence-based guides for culturally-tailored recruitment materials and messaging, and community-informed recruitment plans [21]. The team also was experienced with cancer trial recruitment training for primary care providers [22, 23], community leaders and research staff and quality improvement in recruitment and retention [24]. Additionally, the group had conducted previous research on factors influencing decisions by minorities to participate in research [25], how trust is critical for engagement in research and health care [26], what different populations value in return for their research participation [27], and how the use of community engagement principles and approaches can enhance clinical trial recruitment and retention [28].

The curriculum was influenced by previous training developed by the instructors. Examples include the Meharry-Vanderbilt Community Engaged Research Core “Art of Recruitment” module which was originally created in 2014 and subsequently adapted and tailored for specific trials [29], the curricula developed by the Education Network to Advance Cancer Clinical Trials (ENACCT) to train cancer clinical trial research staff on improving recruitment and retention practices, especially for minority groups [24, 30], and train-the-trainer programs with community leaders to increase cancer clinical trial participation among minority groups [30–35].

The team worked to make sure that the learning materials were up-to-date and supported by research evidence. This was done by reviewing the literature to guide the development of the curriculum, providing documentation of references in the videos where appropriate, and creating compilations of cited references for each learning module for learners to download and review. One of the unique aspects of the course is how it demonstrates the importance of plain language when communicating with patients and potential participants about clinical trials [36]. For example, the course materials were targeted at a fifth to eighth grade reading level to provide a model for how clinical research team members should communicate with potential participants about clinical trials while ensuring the applicability of its content to a diverse group of clinical research team members, including those who do not have a formal advanced education. Additionally, adult learning theory was applied in developing the training, including offering a learning experience that was relevant, self-directed, and problem based [37].

The transdisciplinary working group worked together with videographers to implement the course in Coursera, an online learning platform offering massive open online courses (MOOCs). After implementing a draft version of the course in Coursera, members of the RIC Community Advisory Board (CAB) were asked to provide feedback on the course through online meetings held in December 2018. A key component of the RIC, the 12-member CAB is a racially, ethnically and geographically diverse group, which includes members with experience partnering with academic researchers to engage diverse community members as research participants and research partners. CAB members represent a variety of community-based organizations including health, social service, faith and advocacy and include patients, caregivers, and past clinical trial participants.

The CAB members were given an overview and demonstration of the course, with representative examples of videos, reading assignments, and quizzes provided from Module 1. Discussion questions included if the slides shown in the videos and language were clear, if any visual part of the training hampered the learning experience, if the exercises and quizzes were adequate, and if there was any component they strongly disliked. Notes were taken during the meetings to record the CAB members’ feedback.

Overall, the feedback from the CAB was positive. One recommendation was to modify the suggested answers to the Module 1 in-video quiz questions which addressed how to respond to potential participants’ research concerns and mistrust in researchers and the process due to past research abuses. Specifically, the suggestion was to include language validating the concerns of the potential research participants. The CAB also suggested adding language after the section on historical research abuses in Module 1 to help reassure course participants that steps are being taken to prevent abuses from happening again and providing more examples of why participation in research matters. Modifications to the course were made in response to the feedback received.

The course was titled, “Faster Together, Enhancing the Recruitment of Minorities in Clinical Trials,” and launched on April 1, 2019 in Coursera [38]. Minor changes were made to the reading assignments after the course release date based on feedback from the learners to help clarify the assignments and also to incorporate new references identified after the course release date. The changes that were made did not impact the ability to answer the knowledge assessment used in the course evaluation study. See the results section of the manuscript for a full description of the course.

### Course evaluation study

#### Design, setting, and participants

All participants who enrolled in the course from the course release date, April 1, 2019, through January 31, 2020, were eligible to participate in the course evaluation study described here. Course learners who completed the pre-test were sent an invitation to complete the post-test 1 week later with instructions to begin the survey after completing the course. Responders who completed the post-test by May 17, 2019, had an opportunity to enter a drawing to win one of two $100 Amazon gift cards.

The course was promoted through different mechanisms, including through an online forum for people interested in or having self-described expertise in recruitment, a Trial Innovation Network Collaboration webinar, Medical Library Association poster presentation, and the online Recruitment Innovation Center toolkit. The course was also shared with individual multi-site clinical trials through the Recruitment Innovation Center. Individuals who enrolled in the Coursera course were given brief information about the study and a link to the pre-test through the first reading assignment in the course.

#### Variables and data sources/measures

Pre- and post-test surveys were created using REDCap, a secure online platform for creating and maintaining surveys and databases [39]. The pre-test included basic demographic questions (e.g., race/ethnicity, sex, age, occupation, etc.) and a knowledge assessment consisting of 28 items in multiple choice and true/false formats addressing topics covered in the course (Additional file 2). The pre-test additionally included 10 items addressing participants’ attitudes and intentions regarding recruitment using a 5-point Likert scale based on agreement (Additional file 2) [24].

The post-test included the same knowledge assessment questions and items addressing attitudes and intentions towards minority recruitment as the pre-test. Additionally, participants were asked to indicate whether course participation increased their professional knowledge of the subject (yes/no) and rate how likely they were to make changes in their clinical trial recruitment and retention practices due to course participation using a 4-point Likert scale based on likelihood. Adapted from the ENACCT Pilot Education Program (Additional file 2) [24], 8-items queried participants’ feedback on their training experience.

Data were also collected from the Coursera Course Summary Dashboard [40] on the number of unique visitors to the course website, the number of participants who enrolled in the course, the number of participants who started an item in the course, and the number of participants who completed the course.

#### Study size

The sample size was determined by the responders to the course and the pre- and post-test questionnaires.

#### Data collection

This paper reports data collected through the pre- and post-tests and course enrollment from the Coursera Course Summary Dashboard [40] during the period of course release (April 1, 2019) through January 31, 2020. The pre- and post-tests were expected to require between 15 and 20 min to complete. Participants indicated their willingness to complete the survey by providing written assent.

#### Statistical methods

Frequencies and percentages were used to describe the demographic variables. Because of the small sample size, a paired-sample t-test and Bootstrap confidence intervals were used in 2000 samples to compare pre- and post-assessments of knowledge, attitudes, and intentions as it relates to minority recruitment. Missing data were excluded. Measures of acceptability and satisfaction were summarized using counts and frequencies. There is no allowance for multiplicity. SPSS version 25 was used for all statistical analyses.

## Results

### Course description and features

The intended audience for the online Coursera course *Faster Together, Enhancing the Recruitment of Minorities in Clinical Trials* is individuals currently involved in or seeking to be involved in clinical trial recruitment, including community educators, patient navigators, and research team members. The goal of the course is to provide training and support for researchers and their teams to engage communities not usually approached for research studies as one step toward expanding representation of racial and ethnic minorities in clinical trials. Individuals can enroll in the course at any time and complete the assignments at their own pace. There is no fee to enroll in the course and access the learning materials; however, learners have the option to pay to receive a Coursera course certificate after completing the training. For course completion, learners must achieve a passing score on all graded assignments.

The course is divided into 8 modules: 1) understanding the need to increase minority recruitment in clinical trials; 2) key principles of community engagement; 3) reaching out into the community: effective communication; 4) educating potential research participants; 5) outreach with community healthcare providers; 6) effective screening, education, and decision support; 7) managing an effective, person-centered consent process; 8) person-centered retention. The modules address competencies related to the recruitment of minority populations into research studies and consist of video presentations, reading assignments, and quizzes (Additional file 3). The estimated completion time is approximately 1-h per module, of which the total video duration is 24 min or less per module (Additional file 3).

**Fig. 1:**
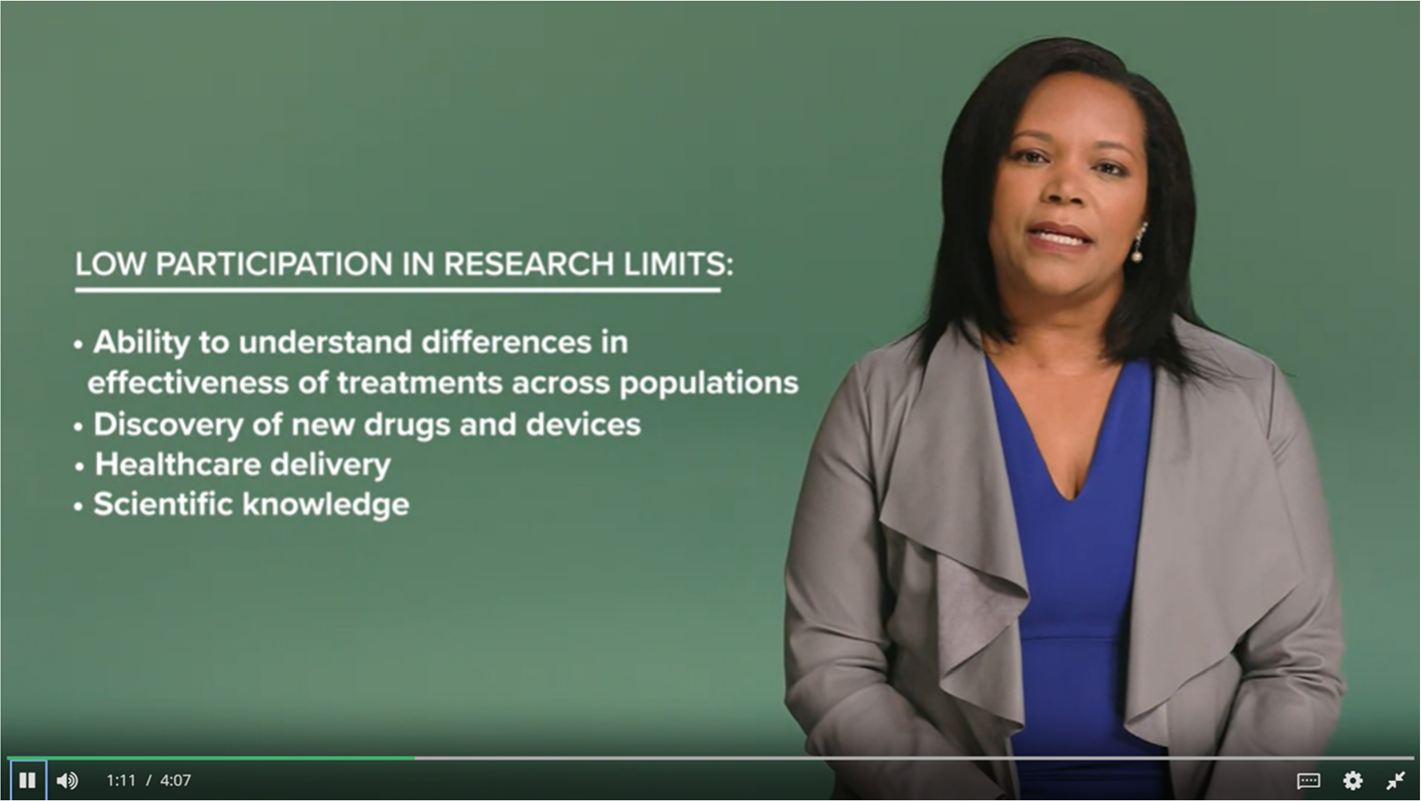
Screenshot of a video from Module 1

The primary didactic content is provided through the video lectures, which feature the course instructors discussing the material, with key points emphasized through text and images, as appropriate (Fig. 1). The course instructors include a community engagement specialist, Executive Director of Community Engagement, clinical trials education specialist, and Vice President of Health Equity- all of whom have extensive years of experience in recruiting underrepresented populations into research. The course instructor videos are supplemented by excerpts of interviews with minority research participants discussing their concerns about participating in clinical research studies. To give learners an opportunity to practice their skills, video scenarios, showing hypothetical interactions between potential participants and research team members, were filmed with actors and inserted in the videos. Learners are asked how they would respond to the different scenarios using an open response format and can view a suggested correct answer after entering a response.

The course includes several unique features to adhere to adult learning principles and foster participatory learning. The reading assignments were designed to supplement the video presentations and include information about freely available toolkits and resources to help research team members create and implement strategies to help increase recruitment and retention of minorities. The module quizzes, which consisted of multiple choice, true/false, and reflective questions, were designed to facilitate the learners’ ability to self-assess their understanding of the learning objectives. The module quizzes are auto-graded, and feedback is provided immediately after learners submit a quiz to stimulate learning. In contrast to the pre- and post-tests, learners can retake the module quizzes until they feel they have completely learned the material. Learners are also asked to complete an action planning tool at the end of each module to help them plan steps they could take to help increase recruitment of minorities in clinical trials at their own institutions. The action planning tool is an optional activity. The modules also included a slide version of the videos and list of references for learners to download from Coursera. The course and its materials can be accessed at http://www.coursera.org/learn/recruitment-minorities-clinical-trials.

### Course effectiveness and reception

From course launch on April 1, 2019 through January 31, 2020, 3382 individuals visited the course website, 382 individuals enrolled in the course, 224 learners started an item in the course, and 46 learners completed the course.

**Fig. 2:**
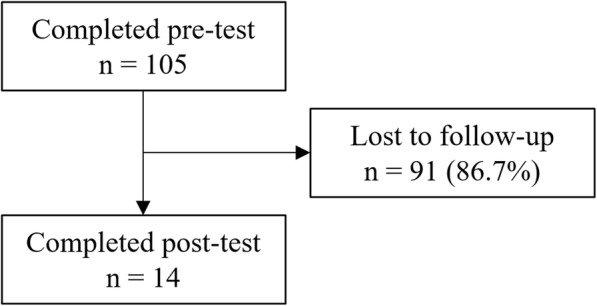
Flowchart of participants in the course evaluation study

One hundred five participants completed the pre-test, and 14 participants completed both the pre- and post-tests (Fig. 2). Baseline characteristics of those who participated in the evaluation study are summarized in Table 1. Of those who completed the pre-test, most respondents were female (85.7%), United States residents (83.7%), and White (55.2%). The median age was 35 years (Interquartile Range [IQR]: 28–47 years).

**Table 1:** Characteristics of respondents to the pre- and post-tests

Category	Completed pre-test (*n =* 105)	Completed post-test (*n* = 14)
Sex, n (%)		
Male	15/105 (14.3%)	2/14 (14.3%)
Female	90/105 (85.7%)	12/14 (85.7%)
United States resident, n (%)		
Yes	87/105 (82.9%)	11/14 (78.6%)
No	17/105 (16.2%)	2/14 (14.3%)
Unknown	1/105 (.952%)	1/14 (7.14%)
Age (LQ^a^-UQ^b^), years	35.00 (28.00–47.00)	31.50 (24.00–37.75)
Race or ethnicity^c^		
African American	19/105 (18.1%)	3/14 (21.4%)
Native American, American Indian, or Alaskan Native	1 (0.95%)	0/14 (0%)
Asian or Pacific Islander	11/105 (10.48%)	4/14 (28.6%)
White	58/105 (55.2%)	7/14 (50.0%)
Latino or Spanish	12/105 (11.4%)	2/14 (14.3%)
Other	3/105 (2.86%)	0/14 (0%)
Prefer not to answer	4/105 (3.81%)	0/14 (0%)
Recruiter		
Yes	61/104 (58.7%)	11/14 (78.6%)
No	43/104 (41.4%)	3/14 (21.4%)
Years involved in recruitment, (LQ^a^-UQ^b^)	2.00 (1.00–5.00)	1.75 (0.88–10.00)
Occupation		
Community health worker	2/105 (1.9%)	0/14 (0%)
Clinical research associate	6/105 (5.71%)	0/14 (0%)
Clinical research coordinator or clinical trial manager	33/105 (31.4%)	5/14 (35.7%)
Physician	7/105 (6.67%)	1/14 (7.14%)
Physician assistant	1/105 (0.95%)	0/14 (0%)
Nurse	4/105 (3.81%)	1/14 (7.14%)
Allied health professional	3/105 (2.86%)	0/14 (0%)
Student	15/105 (14.3%)	3/14 (21.4%)
Other	34/105 (32.4%)	4/14 (28.6%)

Data are proportions (%) or medians (interquartile range). Percentages may not equal 100% due to rounding

^a^*LQ* Lower quartile

^b^*UQ* Upper quartile

^c^Note that participants could select more than one option

The median number of correctly answered pre-test knowledge assessment items for all participants (*N* = 105), participants who completed the pre-test but not the post-test (*n* = 91), and participants who completed both the pre- and post-tests (*n* = 14) was 17.0 items (IQR 13.0–19.0; *N* = 105), 17.0 items (IQR: 13.0–19.0; *n* = 91), and 16.0 items (IQR: 12.8–19.0; *n* = 14), respectively. Changes in knowledge, attitudes, and intentions were assessed using data from participants who completed both the pre- and post-tests (*n* = 14). Participants’ knowledge scores were higher with an increase in the mean number of correct answers from 15.4 (95% CI:12.1–18.7) out of 28 items (55%) on the pre-test to 18.7 (95% CI:17.42–20.2) out of 28 items (66.8%) on the post-test. According to the Bootstrap test, the mean number of correct answers for the pre-test was not within the confidence interval for the post-test (Table 2). The variation of pre- to post- change across the individual knowledge assessment questions is shown in Additional file 4. Performance on individual knowledge assessment questions, reported as the percentage of participants responding correctly, was higher for 21 of the 28 items on the post-test than the pre-test (Additional file 4). Attitudes and intentions for minority recruitment changed very little if at all pre- to post-test. According to the Bootstrap test, the mean scores for attitude and intentions towards minority recruitment on the pre-test were within the confidence interval of the post-test scores (Table 2).

**Table 2:** Comparison of the mean in knowledge, attitudes, and intentions pre-post using paired samples t-test

Item	Pre-test Score Mean (SD)	Post-test Score Mean (SD)	Mean Difference (95% bootstrap CI)
**Knowledge** (# Correct answers out of 28 total)	15.54 (3.55)	18.92 (1.50)	3.39 (1.69 to 5.46)
**Attitudes** (Individual Items, Likert scale)			
I believe that improving clinical trials education with potential participants will improve minority clinical trial accrual	4.85 (0.38)	4.92 (0.28)	0.77 (−0.15 to 0.39)
I believe that improving clinical trials retention practices will improve retention with our participants from ethnic/racial minority groups	4.69 (0.48)	4.92 (0.28)	0.23 (−0.08 to 0.54)
I believe that building relationships with community groups can improve clinical trial participation among ethnic/racial minority groups	4.92 (0.28)	4.92 (0.28)	0.00 (−0.23 to 0.23)
I believe that clinical trial education programs in the community may enhance clinical trials participation among racial and ethnic minority groups.	4.92 (0. 28)	4.92 (0. 28)	0.00 (−0.23 to 0.23)
I believe that better communication with community providers may enhance clinical trials referral.	4.92 (0. 28)	4.92 (0. 28)	0.00 (−0.23 to 0.23)
**Intentions** (Individual Items, Likert scale)			
I intend to work with other members of the team to improve the ways we educate potential participants.	4.77 (0.45)	4.69 (0.48)	−0.08 (− 0.31 to 0.15)
I intend to work with other members of the team to improve our strategies around retention.	4.69 (0.48)	4.69 (0.48)	0.00 (−0.31 to 0.31)
I intend to work with other members of the team to institute new ways to partner with community groups.	4.54 (0.66)	4.77 (0.44)	0.23 (−0.08 to 0.69)
I intend to work with other members of the team to institute new ways to institute clinical trial education programs in the community.	4.54 (0.66)	4.69 (0.48)	0.15 (−0.15 to 0.54)
I intend to work with other members of the team to institute new ways to increase referrals by community providers.	4.38 (0.87)	4.69 (0.48)	0.31 (−0.08 to 0.69)

*SD* Standard deviation, *CI* Confidence interval

All respondents indicated that participating in the course improved their professional knowledge of the subject (14/14; 100%) and that they were either ‘very likely’ (10/14; 71.4%) or ‘somewhat likely’ (4/14; 28.6%) to make changes in their clinical trial recruitment and retention practices as a result of participating in the educational activity. Participants displayed high levels of satisfaction with the learning materials and the course format (Table 3).

**Table 3:** Participant feedback on the course

Questionnaire items and responses	n (%) (***N*** = 14)
Course improved professional knowledge of subject	
Yes	14 (100%)
No	0 (0%)
Likelihood of making changes in clinical trial recruitment and retention practices	
Very likely	10 (71.4%)
Somewhat likely	4 (28.6%)
Somewhat unlikely	0 (0%)
Not at all likely	0 (0%)
Course materials and presentations were free of bias	
Strongly disagree	0 (0%)
Disagree	0 (0%)
Neutral	0 (0%)
Agree	6 (42.9%)
Strongly agree	8 (57.1%)
Learning materials were of high quality	
Strongly disagree	0 (0%)
Disagree	0 (0%)
Neutral	0 (0%)
Agree	7 (50.0%)
Strongly agree	7 (50.0%)
Format was effective in conveying content	
Strongly disagree	0 (0%)
Disagree	0 (0%)
Neutral	0 (0%)
Agree	6 (42.9%)
Strongly agree	8 (57.1%)
Interactive components of the course aided my comprehension of the content	
Strongly disagree	0 (0%)
Disagree	0 (0%)
Neutral	1 (7.1%)
Agree	6 (42.9%)
Strongly agree	7 (50.0%)
Content	
Poor	0 (0%)
Fair	0 (0%)
Average	0 (0%)
Good	6 (42.9%)
Excellent	8 (57.1%)
Provided useful research information	
Poor	0 (0%)
Fair	0 (0%)
Average	0 (0%)
Good	3 (21.4%)
Excellent	11 (78.6%)
Conveyed the subject matter clearly	
Poor	0 (0%)
Fair	0 (0%)
Average	0 (0%)
Good	3 (21.4%)
Excellent	11 (78.6%)
Met learning objectives	
Poor	0 (0%)
Fair	0 (0%)
Average	0 (0%)
Good	4 (28.6%)
Excellent	10 (71.4%)

See Additional file 2 for the full questions and response options

## Discussion

Faster Together is the first reported massive open online course for research team members aimed at improving knowledge and skills to enhance the recruitment of racial and ethnic minorities in clinical trials. Through use of the Coursera platform, the course has the potential to reach a broad audience by offering training in an online, asynchronous learning format. Responses to the course feedback questions indicate learners were satisfied with the course content and format, perceived the course as useful, and were likely to make changes to their recruitment practices as a result of participating in the course. Our evaluation indicates that the course increased participants’ knowledge of the minority recruitment and retention and that learners are motivated to work towards increasing minority recruitment in clinical trials. Although we observed an increase in recruitment and retention knowledge after the course, the intent of this project was to demonstrate the feasibility and acceptability of a MOOC for improving clinical trial recruitment. We did not observe a statistically significant impact of course participation on attitudes and behavioral intentions towards minority recruitment, which may have been the result of ceiling level effects. The overall findings demonstrate that the course has the potential to increase awareness on the importance of and strategies to increase diversity in clinical trials among research teams.

To the best of our knowledge, the course that we developed is the first freely available MOOC on minority recruitment and retention in clinical trials. Through the review of the literature that we conducted to guide the course development process, we saw that there was a lack of comprehensive training available on recruitment of minorities in clinical trials. While we identified several resources that could help research team members, individuals may not be aware that they are available or know how to find them. Participation in the course we developed helps learners gain knowledge and awareness about the importance of including minorities in clinical trials and become familiar with freely available tools and resources that could help them with minority recruitment.

Typical of other MOOCs, the completion rate of individuals who enrolled in the course was low [41–43]. The time commitment required for course completion (approximately 11 h) may be a barrier for many individuals. Offering continuing education credits may help bolster course enrollment and completion. Additional strategies to explore include incorporating either the full course or a subset of the modules as part of required training for research team members.

Limitations of our study include that the pre- and post-tests were not required; therefore, the data does not represent all those who enrolled in and completed the course. While participants were instructed to complete the post-test after finishing the course, they were not asked to show evidence of course completion, such as through submission of a Coursera completion certificate. We chose not to require learners to submit a completion certificate because doing so would have required participants to pay a fee. The knowledge assessment questionnaire was not pre-tested prior to administration in the study; therefore, it is possible that some of the questionnaire items may have been confusing to participants, which could have masked the detection of a greater impact on test performance. We had a small sample which reflected our inability to assess differences in the characteristics of those completing the pre- and post-tests. This may have also contributed to the small impact on knowledge gained and lack of impact on attitudes and intentions observed in our study. Furthermore, there is possible respondent bias, in which participants viewed research positively, leading to potential ceiling effects in our evaluation. We did not assess reasons for why participants did not complete the post-test.

While we assessed changes in knowledge, attitudes and intentions about recruitment after completion of the course, we did not assess the impact of completion of individual modules or measure whether behavior and other outcomes, including minority enrollment in clinical trials, were ultimately altered as a result of participation in the course. We also did not assess whether the changes in knowledge gained after completing the course were long-lasting, which would have required re-administering the knowledge questionnaire several weeks after participants had completed the course.

## Conclusions

There is an urgent need for the research community to ensure that racial and ethnic minorities are adequately represented in clinical research studies. New treatments must be tested in the populations that will need them in order to understand whether there are differences in safety and efficacy. Including diverse populations in research will enable researchers to assess how factors, such as age, sex, race, and lifestyle impact treatment response.

The Faster Together massive open online course helps address a need for comprehensive training on ways to enhance the recruitment and retention of minorities in clinical trials as one step towards ending inequitable representation in research. Future work is needed to explore ways to increase course enrollment and completion within the clinical research community. Additionally, formal assessments should be conducted to determine whether completion of the training course is associated with improvements in accrual of racial and ethnic minorities in clinical trials.

## Supplementary Information

**Additional file 1.** Methods and results of literature review on knowledge needed to enhance minority recruitment and existing training programs and resources on minority recruitment.

**Additional file 2.** Questionnaire items addressing knowledge, attitudes and intentions, and feedback on the course.

**Additional file 3.** Course overview: core competencies, learning objectives, and assignments.

**Additional file 4.** Percentage of participants who completed both the pre- and post-tests (*N* = 14), who responded correctly to individual items on the knowledge assessment.

## Data Availability

The datasets used during the current study are available from the corresponding author on reasonable request.

## Abbreviations

CAB: Community Advisory Board
ENACCT: Education Network to Advance Cancer Clinical Trials
EMPaCT: Enhancing Minority Participation in Clinical Trials
IRB: Institutional Review Board
IQR: Interquartile Range
MOOC: Massive open online course
NIH: National Institutes of Health
RIC: Recruitment Innovation Center
TIN: Trials Innovation Network
