# Supplementary material for: Design and implementation of a massive open online course on enhancing the recruitment of minorities in clinical trials – Faster Together
Source: BMC Med Res Methodol. 2021 Mar 5;21:44. doi: 10.1186/s12874-021-01240-x (PMC7936494; doi:10.1186/s12874-021-01240-x)
Supplement: Supplementary file 1 — Additional file 1. Methods and results of literature review on knowledge needed to enhance minority recruitment and existing training programs and resources on minority recruitment. [file 12874_2021_1240_MOESM1_ESM.docx]

**Additional file 1.** Methods and results of literature review on knowledge needed to enhance minority recruitment and existing training programs and resources on minority recruitment

METHODS

A search of PubMed, Web of Science, and the Cumulative Index to Nursing and Allied Health Literature (CINAHL) databases and the grey literature was conducted to identify 1) literature addressing the knowledge and skills recruiters, research coordinators and research teams’ need to gain to effectively recruit and retain minorities in clinical trials and 2) existing training programs and resources available to enhance recruiters, research coordinators and research teams’ ability to recruit and retain minorities in clinical trials. The literature review was conducted in the early stages of the course development process and included information identified until September 29, 2017.

Terminology for three distinct concepts was developed and formatted for the three search platforms (PubMed, Web of Science, and CINAHL). Concept 1 included the terms patient selection; patient dropouts; crowdsourcing; recruitment; recruit; enrolling; enrollment; enrolment; research personnel; clinical trial; clinical trials; trial recruitment; research subject recruitment. Concept 2 included the terms cultural competency; cultural competence; training; education; course; class; education; workshop; culturally tailored; culturally sensitive; culturally appropriate; cultural humility; culturally mindful; mindful; cultural tradition; cultural sensitivity; cultural diversity. Concept 3 included the terms minority; minorities; underrepresented; under-represented; under served; disenfranchised; under-enrollment; African American; African Americans; Hispanic; Hispanics; Hispanic Americans; Hispanic American. The concept groups were combined in multiple patterns to search the three databases. In addition, cited reference searching was conducted on Web of Science for selected papers. Grey literature was identified through searches of Google and by searching websites of relevant organizations.

RESULTS

**Knowledge and Skills Needed for Effective Recruitment of Minorities**

Studies that have reported on the training needs of researchers and staff include a qualitative study reporting findings from interviews with principal investigators, research staff, referring clinicians, and cancer center leaders at five U.S. cancer centers affiliated with the consortium for Enhancing Minority Participation in Clinical Trials,^1^ a study reporting perspectives of community members from underrepresented populations,^2^ a report from the NRG Oncology Workshop on challenges in clinical trial enrollment,^3^ a review discussing ways to increase recruitment of minorities in clinical trials,^4^ and a journal article targeting family practitioners reporting ways to increase minority participation in research.^5^

Based on the reported literature, knowledge and skills that may be needed to improve recruitment and retention of minorities in clinical trials include an understanding of factors affecting minority participation in research^1^ and awareness of the importance of appropriate communication and establishing patient trust.^3^ The literature suggests training in cultural awareness, cultural competence, and humility and training regarding ways to improve communication with minorities is needed.^1,2,4^ Additionally, investigators must understand characteristics of the population they are planning to recruit and be aware of provider-related barriers and the need to establish trust with referring physicians.^5^

**Training and Resources on Recruitment and Retention of Minorities in Clinical Trials**

Several training programs and resources on recruitment and retention of minorities in clinical trials were identified through the literature review (see table below).^6-32^ Focus topics include awareness of the need for diversity in clinical trials, awareness of barriers affecting minority populations, awareness of personal biases, cultural competency, culturally appropriate methods for increasing recruitment and retention, successful models for minority recruitment, building trust, establishing rapport, patient engagement, increasing awareness of clinical trials among minorities, educating minorities about clinical trials, assessing the effectiveness of minority recruitment, reimbursement and IRB regulations, and developing informed consents for limited English-speaking, low health literacy/numeracy participants. The agencies offering training include government, non-profit organizations, and universities. Five of the online training courses that were originally identified in 2017 were only available through the Web archive three years later, at the time of manuscript writing.^6-10^

**Table. Programs and resources on recruitment and retention of minorities in clinical trials:**

| **Name of program or resource [reference]** | **Organization** | **Format** |
| --- | --- | --- |
| Building Trust and Establishing Rapport [6] | Enhancing Minority Participation in Clinical Trials (EMPaCT) | Online training course |
| Successful Models for Minority Recruitment [7] | EMPaCT | Online training course |
| Cultural Competency Training [8] | EMPaCT | Online training course |
| Increasing Clinical Trial Awareness Among Minorities [9] | EMPaCT | Online training course |
| Assessing Effectiveness of Minority Recruitment Efforts [10] | EMPaCT | Online training course |
| Diversity Awareness for Sites & Sponsors [11] | TransCelerate BioPharma Inc. | Webinar |
| Patient Engagement [12] | TransCelerate BioPharma Inc. | Webinar |
| Cultural Competency [13] | TransCelerate BioPharma Inc. | Webinar |
| Reimbursement IRB Insights [14] | TransCelerate BioPharma Inc. | Webinar |
| Informed Consent Short Form [15] | TransCelerate BioPharma Inc. | Webinar |
| Community Engagement for Sites [16] | TransCelerate BioPharma Inc. | Webinar |
| Cultural Competency Training and Recruitment Training Program [17] | NRG Oncology Radiation Therapy Oncology Group  June 2011 RTOG semi-annual meeting in Philadelphia, PA | In-person training |
| Practical Tips to Improve Asian American Participation in Cancer Clinical Trials [18] | Stanford | Online course |
| Reshaping Research: A Guide to Enhancing Cultural Considerations into Research. Module 4: Recruitment and Retention [19] | Center for Reducing Health Disparities, Case Western Reserve University | Guide |
| Unconscious Bias: An Introduction [20] | Case Western Reserve University | Text document |
| Health Care Provider Toolkit [21] | Clinical Trial Engagement Network | Toolkit |
| Points to Consider about Recruitment and Retention While Preparing a Clinical Research Study [22] | National Institute of Mental Health | Text document |
| Outreach Notebook for the Inclusion, Recruitment and Retention of Women and Minority Subjects in Clinical Research [23] | National Institutes of Health | Principal investigators’ notebook |
| Cancer Clinical Trials A Resource Guide for Outreach, Education, and Advocacy [24] | National Institutes of Health, National Cancer Institute | Booklet/pamphlet |
| Distrust, Race, and Research: Overcoming Barriers to Recruitment and Retention of Minority Populations [25] | Public Responsibility in Medicine and Research (PRIM&R) | Webinar |
| Cultural Competence in Research [26] | The Harvard Clinical and Translational Science Center | Annotated bibliography |
| Increasing Minority Participation in Clinical Research [27] | The Endocrine Society | White paper |
| Self-assessment of cultural attitudes and competence of clinical investigators to enhance recruitment and participation of minority populations in research [28] | Creighton University, Center for Health Policy and Ethics | Journal article |
| Responding to Culture: Beyond Cultural Competence Training [29] | Robert Wood Johnson Foundation | Online publication |
| Communities as Partners in Cancer Clinical Trials: Changing Research, Practice and Policy [30] | Education Network to Advance Cancer Clinical Trials (ENACCT) | Online publication |
| The Importance of Communication and Cultural Competence in Enhancing Research Participant Recruitment and Retention [31] | Johns Hopkins University School of Medicine | Slides |
| Culture-Driven Patient Recruitment and Retention [32] | The Association of Clinical Research Professionals (ACRP) | Required ACRP membership to access |

REFERENCES

1. Niranjan SJ, Durant RW, Wenzel JA, Cook ED, Fouad MN, Vickers SM, et al. Training needs of clinical and research professionals to optimize minority recruitment and retention in cancer clinical trials. J Cancer Educ. 2019;34:26-34.
2. Erves JC, Mayo-Gamble TL, Malin-Fair A, Boyer A, Joosten Y, Vaughn YC, et al. Needs, priorities, and recommendations for engaging underrepresented populations in clinical research: A community perspective. J Community Health. 2017;42:472-480.
3. Brooks SE, Muller CY, Robinson W, Walker EM, Yeager K, Cook ED, et al. Increasing Minority Enrollment Onto Clinical Trials: Practical Strategies and Challenges Emerge From the NRG Oncology Accrual Workshop. J Oncol Pract. 2015;11:486-90.
4. Salman A, Nguyen C, Lee YH, Cooksey-James T. A Review of Barriers to Minorities' Participation in Cancer Clinical Trials: Implications for Future Cancer Research. J Immigr Minor Health 2016;18:447-53.
5. Diaz V. Encouraging participation of minorities in research studies. Ann Fam Med. 2012;10: 372-3.
6. Jones LA, Hawk E, Hurd TC, Hoang TV, Lewis AJ, Berglund MR, et al. Building Trust and Establishing Rapport. Minneapolis (MN): University of Minnesota, EMPACT [cited 2020 Apr 09]. [http://web.archive.org/web/20170630054243/http://empactconsortium.com/training-course/building-rapport-and-establishing-trust/](about:blank). Accessed 9 April 2020.
7. Fouad M, Durant R, Martin M. Successful models for minority recruitment. Minneapolis (MN): University of Minnesota, EMPACT. [http://web.archive.org/web/20170630053647/http://empactconsortium.com/training-course/successful-models-for-minority-recruitment/](about:blank). Accessed 9 April 2020.
8. Chen MS, Dang J, Nguyen TT, Ton D, Fung T. Cultural Competency Training. Minneapolis (MN): University of Minnesota, EMPACT. [http://web.archive.org/web/20170630050815/http://empactconsortium.com/training-course/cultural-competency-training/](about:blank). Accessed 9 April 2020.
9. Ford JG, Wenzel J, Mgah O. Increasing clinical trial awareness among minorities. Minneapolis (MN): University of Minnesota, EMPACT. [http://web.archive.org/web/20170630052640/http://empactconsortium.com/training-course/increasing-clinical-trial-awareness-among-minorities/](about:blank). Accessed 9 April 2020.
10. Vickers SM, Hawk E, Ghebre R, Habermann E, Rogers LM. Assessing effectiveness of minority recruitment efforts. Minneapolis (MN): University of Minnesota, EMPACT. [http://web.archive.org/web/20170630052544/http:/empactconsortium.com/training-course/assessing-effectiveness-of-minority-recruitment-efforts/](about:blank). Accessed 9 April 2020.
11. TransCelerate Biopharma Inc. Diversity Awareness for Sites & Sponsors. [https://transceleratebiopharmainc.com/assets/clinical-trial-diversification-4/](about:blank). Accessed 2020 Feb 21.
12. TransCelerate Biopharma Inc. Patient Engagement. [https://transceleratebiopharmainc.com/assets/clinical-trial-diversification-4/](about:blank). Accessed 2020 Feb 21.
13. TransCelerate Biopharma Inc. Cultural Competency. [https://transceleratebiopharmainc.com/assets/clinical-trial-diversification-4/](about:blank). Accessed 2020 Feb 21.
14. TransCelerate Biopharma Inc. Reimbursement IRB Insights. [https://transceleratebiopharmainc.com/assets/clinical-trial-diversification-4/](about:blank). Accessed 2020 Feb 21.
15. TransCelerate Biopharma Inc. Informed Consent Short Form. [https://transceleratebiopharmainc.com/assets/clinical-trial-diversification-4/](about:blank). Accessed 2020 Feb 21.
16. TransCelerate Biopharma Inc. Community Engagement for Sites. [https://transceleratebiopharmainc.com/assets/clinical-trial-diversification-4/](about:blank). Accessed 2020 Feb 21.
17. Wells JS, Pugh S, Boparai K, Rearden J, Yeager KA, Bruner DW. Cultural competency training to increase minority enrollment into radiation therapy clinical trials-an NRG oncology RTOG study. J Cancer Educ. 2017;32:721-727.
18. Stanford Center for Continuing Medical Education. Practical Tips to Improve Asian American Participation in Cancer Clinical Trials. 2015. [https://med.stanford.edu/cme/courses/online/clinical-trials.html](about:blank). Accessed 2020 Feb 21.
19. Case Western Reserve University. Center for Reducing Health Disparities. Reshaping research: A guide to enhancing cultural considerations into research. Module 4: Recruitment and Retention. https://case.edu/research/sites/case.edu.research/files/2018-04/module-4-handout-from-CRHD.pdf. Accessed 2020 April 29.
20. Case Western Reserve University. Case Comprehensive Cancer Center. Unconscious Bias: An Introduction. 2015. [https://case.edu/cancer/research/clinical-research-office/minority-accrual-committee/cultural-inclusion-and-awareness/unconscious-bias-introduction](about:blank). Accessed 2020 Feb 21.
21. Clinical Trial Engagement Network. Health Care Provider Toolkit. [Internet]. [cited 2017 Sep 29]. [https://www.joinimin.org/uploads/documents/Im_In_Physician_Toolkit.pdf](about:blank). Accessed 2017 Sep 29.
22. National Institute of Mental Health. Points to consider about recruitment and retention while preparing a clinical research study. [https://www.nimh.nih.gov/funding/grant-writing-and-application-process/recruitment-points-to-consider-6-1-05_34848.pdf](about:blank). Accessed 2020 Feb 21.
23. National Institutes of Health. Outreach notebook for the inclusion, recruitment and retention of women and minority subjects in clinical research. [http://www.chicagoamwa.org/uploads/2/8/7/0/28704181/outreach-notebook-2015.pdf](about:blank). Accessed 2020 Feb 21.
24. National Institutes of Health. National Cancer Institute. Cancer clinical trials: a resource guide for outreach, education and advocacy. [https://pubs.cancer.gov/ncipl/detail.aspx?prodid=P921](about:blank). Accessed 2020 Feb 21.
25. Public Responsibility in Medicine and Research. Distrust, race and research: overcoming barriers to recruitment and retention of minority populations. 2010. [https://www.primr.org/webinars/2010/distrust/](about:blank). Accessed 2020 Feb 21.
26. Harvard Catalyst. The Harvard Clinical and Translational Science Center. Cultural competence in research. Program for Faculty Development & Diversity. Monitoring, Evaluation, Accountability and Learning (MEAL). The Open University, USA, 2010. [https://catalyst.harvard.edu/pdf/diversity/CCR-annotated-bibliography-10-12-10ver2-FINAL.pdf](about:blank). Accessed 2020 Feb 21.
27. Agodoa, Lawrence, Alfonso J. Alanis, Maria Alexander-Bridges, Loretta L. Doan, G. Alexander Fleming, Ken Getz, Brian K. Gibbs, and Deborah Prothrow-Stith. Increasing minority participation in clinical research. 2007. [https://sph.umd.edu/sites/default/files/files/http___www_hormone_org_Public_clinical_trials_content_loader.pdf](about:blank). Accessed 2020 Feb 21.
28. O'Brien RL, Kosoko-Lasaki O, Cook CT, Kissell J, Peak F, Williams EH. Self-assessment of cultural attitudes and competence of clinical investigators to enhance recruitment and participation of minority populations in research. J Natl Med Assoc. 98, 674-82. (2006).
29. Voss-DeMeester RH, McCullough KW, Cook SC, El-Shamaa M, Chin MH. Robert Wood Johnson Foundation. Responding to Culture: Beyond Cultural Competence Training. [https://www.solvingdisparities.org/sites/default/files/FindingAnswers_RespondingToCulture_0.pdf](about:blank). Accessed 2020 Feb 21.
30. Education Network to Advance Cancer Clinical Trials (ENACCT). Communities as partners in cancer clinical trials: changing research, practice and policy. [http://researchtoolkit.org.isomedia.net/media/content/ENACCT%20Report-Communities%20As%20Partners.pdf](about:blank). Accessed 2020 Apr 09.
31. Cooper LA. The importance of communication and cultural competence in enhancing research participant recruitment and retention. [http://ictr.johnshopkins.edu/wp-content/uploads/import/871-L%20Cooper%20Plenary%20Speaker.pdf](about:blank). Accessed 2020 Feb 21.
32. The Association of Clinical Research Professionals. Culture-Driven Patient Recruitment and Retention. 2016. [https://acrpnet.org/2016/08/01/culture-driven-patient-recruitment-retention/](about:blank). Accessed 2020 Feb 21.
