# Supplementary material for: Design and implementation of a massive open online course on enhancing the recruitment of minorities in clinical trials – Faster Together
Source: BMC Med Res Methodol. 2021 Mar 5;21:44. doi: 10.1186/s12874-021-01240-x (PMC7936494; doi:10.1186/s12874-021-01240-x)
Supplement: Supplementary file 2 — Additional file 2. Questionnaire items addressing knowledge, attitudes and intentions, and feedback on the course. [file 12874_2021_1240_MOESM2_ESM.docx]

**Additional file 2.** Questionnaire items addressing knowledge, attitudes and intentions, and feedback on the course

**Knowledge Assessment (Pre- and Post-test)**

1. Why is it important to increase minority recruitment to clinical trials?
   1. So we can better understand differences in effectiveness of new treatments across populations
   2. So we can more quickly discover new drugs and devices
   3. So we can improve healthcare delivery and health disparities
   4. All of these
2. Why is research participation so low among people from racial or ethnic minority groups?
   1. They tend not to be offered the opportunity to participate, even when they appear to be eligible
   2. They may not be aware of clinical research
   3. They may not qualify for trials because of restrictive eligibility criteria
   4. They tend to be unwilling to participate in research
   5. A, b and d
   6. A, b and c
3. Which of the following is NOT true about biases towards potential research participants?
   1. We all have biases that we are unaware of, they are hard to control, and they can affect many ways we interact with participants from ethnic and racial groups
   2. Those working in research have no real biases; we treat everyone equally
4. Why should research teams work with community groups to improve minority participation in clinical trials?
   1. They can help develop recruitment strategies
   2. They can assist with creating research materials
   3. They can identify best channels for communication with potential participants
   4. They can ensure their members participate in the study
   5. A, b and d
   6. A, b and c
5. Which of the following are guiding principles when working and engaging with community partners around research studies?
   1. Humility, trust, respect, finding mutual benefit, making sure there is a power balance, and transparency
   2. Humility, trust, respect, providing financial incentives and hiring research staff from the community
   3. Humility, trust, transparency, providing trinkets as incentives for recruitment, and training community leaders
6. When learning about your community (your audience) which of the following IS NOT important to consider?
   1. Addressing the needs of adult learners
   2. Assessing and addressing their understanding of clinical trials
   3. Considering health literacy, literacy and numeracy skills
   4. Assessing their eligibility for the trial
   5. Learning about culture
7. What are some ways of tailoring clinical trial messages?
   1. Using plain language, checking the readability of your materials, translating your materials and keeping it simple
   2. Using plain language, checking the readability of your materials, testing your materials and keeping it simple
   3. Using plain language, translating your materials and keeping it simple
8. According to data from National Assessment of Adult Literacy, about what percentage of your potential trial participants would have proficient heath literacy skills?
   1. 5%
   2. 12%
   3. 20%
   4. 75%
   5. More than 80%
9. What does "Return of Value" mean when it comes to working with communities around clinical trials?
   1. Researchers share the information on the study to participants and communities around research results.
   2. Researchers foster greater awareness of the impact that clinical trials can have and the value of the community's participation.
   3. Researchers inform communities about where study results have been published
   4. All of these
   5. A and b
10. The way we educate potential participants about clinical trials should vary according to their health status AND their existing knowledge, attitudes and beliefs about clinical trials
    1. True
    2. False
11. The purpose of clinical trials education is to encourage potential enrollment in a trial
    1. True
    2. False
12. Clinical trial educational materials should provide sufficient information around randomization, the use of placebos and the consent process
    1. True
    2. False
13. It is important to address an individual's culture in clinical trials education; this generally refers to someone's race or ethnicity
    1. True
    2. False
14. Which of the following is NOT TRUE about engaging community-based physicians to help improve minority participation in clinical trials
    1. The research team needs to build trust and understand provider needs and preferences about clinical trials.
    2. The research team needs to engage providers early on in the trial
    3. A research team member needs to be embedded in the community clinic
    4. The research team needs to consider what providers need to get the buy in from their patients
15. Why is it important to engage community-based physicians for the improvement of minority recruitment in clinical trials?
    1. Their patients trust them and their endorsement around a clinical trial
    2. Their patient panel may be mostly ethnic and/or racial minorities
    3. Their patients are often suffering from a number of illnesses being studied in clinical trials
16. Which of the following is NOT true?
    1. Prescreening is defined as matching "basic" patient information to clinical trial eligibility criteria
    2. Systematic pre-screening of potential trial participants has been shown to reduce physician burden and improve clinical trial accrual rates.
    3. Studies have shown that 1/3 or more of sites have no systematic pre-screening process
    4. Prescreening has been shown to bias potential trial participants from minority groups
17. Teaching a potential participant about clinical trials is optimally done during the consent process
    1. True
    2. False
18. Fostering informed decision making is a critical part of pre-consent education
    1. True
    2. False
19. The purpose of educating potential participants is to have them sign a consent form
    1. True
    2. False
20. Which of the following should not be addressed as part of person-centered clinical trial education prior to consent?
    1. Participant's culture and cultural values
    2. Participant's learning style
    3. Participant's insurance coverage
    4. Participant's emotional state
    5. Participant's literacy/health literacy
21. "Person-centered care" has little relevance in clinical trials education or in the consent process
    1. True
    2. False
22. Reading a consent form aloud is a good way to ensure comprehension, especially for participants from ethnic or racial minority groups
    1. True
    2. False
23. What is the best way to ensure comprehension during the consent process?
    1. Making eye contact with the participant
    2. Asking frequently "Do you understand" or "Do you have any questions?
    3. Using the "teach-back" approach
    4. Reading the consent form aloud and stopping to see if they have questions
24. Which of the following policies/procedures are important for your site to ensure it conducts a person-centered consent process?
    1. Addressing the needs of participants with Limited English Proficiency
    2. Ensuring that all pages are signed appropriately
    3. Ensuring informed decision-making
    4. Assessing comprehension throughout the consent process
    5. A, c and d
    6. A, b and d
25. Which of these concepts require extra care in explaining during the consent process, as they are often confusing to potential participants?
    1. Randomization, placebo use, potential benefits and risks, therapeutic misconception
    2. Randomization, placebo use, potential benefits and risks, trial purpose
    3. Randomization, placebo use, potential benefits and risks, sticking to study requirements
    4. Randomization, placebo use, potential benefits and risks, physician as investigator
26. Participant dropout rates in phase 3 clinical trials can often be very substantial, sometimes more than 50%.
    1. True
    2. False
27. Which of the following barriers have been shown to contribute to lower retention rates for participants from ethnic and racial minority groups?
    1. Transportation barriers
    2. Mistrust of research staff
    3. Side effects
    4. Family and work obligations
    5. A, b and c
    6. A, b and d
28. Which of the following strategies has been suggested to improve retention rates for participants from ethnic and racial minority groups?
    1. Relationship building between participants and staff
    2. Providing incentives
    3. Setting up a community advisory board
    4. Visiting participants at home
    5. A, b and c
    6. A, b and d

**Beliefs and Intentions about Minority Recruitment (Pre- and Post-test)**

On a scale of 1 (Strongly Disagree) to 5 (Strongly Agree), please indicate your level of agreement with the following statements.

1. I believe that improving clinical trials education with potential participants will improve minority clinical trial accrual
2. I intend to work with other members of the team to improve the ways we educate potential participants.
3. I believe that improving clinical trials retention practices will improve retention with our participants from ethnic/racial minority groups
4. I intend to work with other members of the team to improve our strategies around retention.
5. I believe that building relationships with community groups can improve clinical trial participation among ethnic/racial minority groups
6. I intend to work with other members of the team to institute new ways to partner with community groups.
7. I believe that clinical trial education programs in the community may enhance clinical trials participation among racial and ethnic minority groups.
8. I intend to work with other members of the team to institute new ways to institute clinical trial education programs in the community.
9. I believe that better communication with community providers may enhance clinical trials referral.
10. I intend to work with other members of the team to institute new ways to increase referrals by community providers.

**Course feedback (Post-test)**

1. Did participating in this course increase your professional knowledge of the subject? (yes/no)
2. As a result of participating in this educational activity, how likely are you to make changes in your clinical trial recruitment and retention practices? (Not at all likely, somewhat unlikely, somewhat likely, very likely)

On a scale of 1 (strongly disagree) to 5 (strongly agree), please indicate your level of agreement with the following statements

1. Course materials and presentations were free of bias
2. Learning materials were of high quality
3. Format was effective in conveying content
4. Interactive components of the course aided my comprehension of the content

On a scale of 1 (poor) to 5 (excellent), please rate the following aspects of the lessons.

1. Content
2. Provided useful research information
3. Conveyed the subject matter clearly
4. Met learning objectives

If you rated any of the above items as poor or fair, please outline your concern.

Please provide general comments about the course.
