# Supplementary material for: Design and implementation of a massive open online course on enhancing the recruitment of minorities in clinical trials – Faster Together
Source: BMC Med Res Methodol. 2021 Mar 5;21:44. doi: 10.1186/s12874-021-01240-x (PMC7936494; doi:10.1186/s12874-021-01240-x)
Supplement: Supplementary file 3 — Additional file 3. Course overview: core competencies, learning objectives, and assignments. [file 12874_2021_1240_MOESM3_ESM.docx]

**Additional file 3. Course Overview: Core Competencies, Learning Objectives, and Assignments**

| Module | Title | Core Competency | Learning Objectives | #Videos, reading assignments, quizzes | Total Video Length |
| --- | --- | --- | --- | --- | --- |
| 1 | Understanding the need to increase minority recruitment in clinical trials | The aim of this module is to provide recruiters with tools to advocate for increasing minority participation in clinical trials. | - Describe the impact of low minority participation in clinical research on medical care. - Name key examples of unethical research. - Identify barriers and facilitators to participation in clinical trials for minority groups. | 5 videos, 6 readings, 2 quizzes | 23 minutes |
| 2 | Key principles of community engagement | The aim of this module is to provide tools to recruiters to increase minority participation in clinical trials through the use of person-centered health education practices and communication skills that include cultural humility and rapport-building with individuals, families, providers and community groups throughout outreach, recruitment, accrual and retention processes. | - Examine one's ability to relate to diverse populations and the impact of bias. - Describe the key principles of community engagement. - Explain the critical steps to building effective/sustainable community partnerships. - Identify how community partnerships can inform, enhance, and strengthen recruitment strategies. | 3 videos, 6 readings, 1 quiz | 22 minutes |
| 3 | Reaching out into the community: effective communication | The aim of this module is to provide tools to recruiters to manage external trial promotion communication and partnership efforts within the community (outreach), using the principles of community engagement, person-centered health education practices and communication skills and the principles of return of value to partnered groups. | - Describe four ways to improve communication with the community about clinical trials. - List ways to tailor your message to your audience. - List readability tools and clear communication guidelines to consult when developing written information about clinical trials for lay audiences. - Apply principles of plain language and cultural appropriateness to create print or digital study materials. | 4 videos, 5 readings, 1 quiz | 24 minutes |
| 4 | Educating potential research participants | The aim of this module is to provide tools to recruiters to use person-centered communication skills and health education practices for both oral and written communication, including plain language, cultural and linguistic appropriate standards and understanding behavior change. | - Describe the purpose of educating potential participants about clinical trials. - Describe ways to build interest in joining a study. - Describe ways to tailor education about clinical trials for participants. | 3 videos, 4 readings, 1 quiz | 15 minutes |
| 5 | Outreach with community healthcare providers | The aim of this module is to provide tools to recruiters to manage external trial promotion and communication efforts with health care providers seeing significant percentage of minority patients, to use person-centered health education principles and communication skills, to apply the principles of community engagement and of return of value to groups with whom we partner. | - Explain the importance of encouraging provider referrals to clinical trials. - Describe ways to encourage provider referral to clinical trials. | 1 video, 3 readings, 1 quiz | 12 minutes |
| 6 | Effective screening, education, and decision support | The aim of this module is to provide knowledge and tools to recruiters to manage efficient pre-screening, screening and enrollment in clinic or community settings, to use person-centered communication skills and health education practices. | - Describe how systematic screening can make a difference in accrual. - Explain how "person-centered care" can and should be addressed in the context of clinical trials education. - Describe the five components of pre-consent education. | 4 videos, 3 readings, 1 quiz | 19 minutes |
| 7 | Managing an effective, person-centered consent process | The aim of this module is to provide tools to recruiters to manage effective, person-centered consent process, using person-centered communication skills and health education practices | - Describe key components of person-centered consent. - Describe ways to address the information needs of potential participants with low literacy or low health literacy during the informed consent process. - Describe ways to assess participant understanding during the informed consent process. | 3 videos, 6 readings, 1 quiz | 23 minutes |
| 8 | Person-centered retention | The aim of this module is to provide tools to recruiters to manage effective, person-centered compliance and retention strategies, to use person-centered communication skills and health education practices | - Explain why participant retention in clinical trials is important. - Describe common retention challenges for participants from ethnic or racial minority groups. - Describe promising approaches for improving retention. | 1 video, 5 readings, 1 quiz | 7 minutes |
