# Supplementary material for: Design and implementation of a massive open online course on enhancing the recruitment of minorities in clinical trials – Faster Together
Source: BMC Med Res Methodol. 2021 Mar 5;21:44. doi: 10.1186/s12874-021-01240-x (PMC7936494; doi:10.1186/s12874-021-01240-x)
Supplement: Supplementary file 4 — Additional file 4. Percentage of participants who completed both the pre- and post-tests (N = 14), who responded correctly to individual items on the knowledge assessment. [file 12874_2021_1240_MOESM4_ESM.docx]

**Additional file 4.** Percentage of participants who completed both the pre- and post-tests (N=14), who responded correctly to individual items on the knowledge assessment

| Item # | Question | Module | Pre-test | Post-test | Change in percentage |
| --- | --- | --- | --- | --- | --- |
| 1 | Why is it important to increase minority recruitment to clinical trials? | 1 | 78.6 | 93.0 | 14.3 |
| 2 | Why is research participation so low among people from racial or ethnic minority groups? | 1 | 42.9 | 57.1 | 14.3 |
| 3 | Which of the following is NOT true about biases towards potential research participants? | 2 | 35.7 | 42.9 | 7.14 |
| 4 | Why should research teams work with community groups to improve minority participation in clinical trials? | 2 | 85.7 | 100 | 14.3 |
| 5 | Which of the following are guiding principles when working and engaging with community partners around research studies? | 2 | 92.9 | 92.9 | 0 |
| 6 | When learning about your community (your audience) which of the following is NOT important to consider? | 3 | 71.4 | 100 | 28.6 |
| 7 | What are some ways of tailoring clinical trial messages? | 3 | 35.7 | 57.1 | 21.4 |
| 8 | According to data from National Assessment of Adult Literacy, about what percentage of your potential trial participants would have proficient health literacy skills? | 3 | 0 | 0 | 0 |
| 9 | What does “Return of Value” mean when it comes to working with communities around clinical trials? | 3 | 14.3 | 14.3 | 0 |
| 10 | The way we educate potential participants about clinical trials should vary according to their health status AND their existing knowledge, attitudes and beliefs about clinical trials | 4 | 71.4 | 92.9 | 21.4 |
| 11 | The purpose of clinical trials education is to encourage potential enrollment in a trial | 4 | 42.9 | 57.1 | 14.3 |
| 12 | Clinical trial educational materials should provide sufficient information around randomization, the use of placebos and the consent process | 4 | 7.14 | 7.14 | 0 |
| 13 | It is important to address an individual’s culture in clinical trials education; this generally refers to someone’s race or ethnicity | 4 | 14.3 | 28.6 | 14.3 |
| 14 | Which of the following is NOT TRUE about engaging community-based physicians to help improve minority participation in clinical trials | 5 | 28.6 | 42.9 | 14.3 |
| 15 | Why is it important to engage community-based physicians for the improvement of minority recruitment in clinical trials? | 5 | 78.6 | 100 | 21.4 |
| 16 | Which of the following is NOT true? | 6 | 35.7 | 57.1 | 21.4 |
| 17 | Teaching a potential participant about clinical trials is optimally done during the consent process | 6 | 57.1 | 85.7 | 28.6 |
| 18 | Fostering informed decision making is a critical part of pre-consent education | 6 | 92.9 | 100 | 7.14 |
| 19 | The purpose of educating potential participants is to have them sign a consent form | 6 | 92.9 | 100 | 7.14 |
| 20 | Which of the following should not be addressed as part of person-centered clinical trial education prior to consent? | 6 | 78.6 | 92.9 | 14.3 |
| 21 | “Person-centered care” has little relevance in clinical trials education or in the consent process | 7 | 92.9 | 100 | 7.14 |
| 22 | Reading a consent form aloud is a good way to ensure comprehension, especially for participants from ethnic or racial minority groups | 7 | 35.7 | 35.7 | 0 |
| 23 | What is the best way to ensure comprehension during the consent process? | 7 | 64.3 | 100 | 35.7 |
| 24 | Which of the following policies/procedures are important for your site to ensure it conducts a person-centered consent process? | 7 | 85.7 | 78.6 | -7.14 |
| 25 | Which of these concepts require extra care in explaining during the consent process, as they are often confusing to potential participants? | 7 | 50 | 57.1 | 7.14 |
| 26 | Participant dropout rates in phase 3 clinical trials can often be very substantial, sometimes more than 50% | 8 | 7.14 | 21.4 | 14.3 |
| 27 | Which of the following barriers have been shown to contribute to lower retention rates for participants from ethnic and racial minority groups? | 8 | 78.6 | 100 | 21.4 |
| 28 | Which of the following strategies has been suggested to improve retention rates for participants from ethnic and racial minority groups? | 8 | 71.4 | 57.1 | -14.3 |

See Additional file 2 for the full questions and response options in the knowledge assessment.
